# SELF-IDENTIFIED STRENGTHS AMONG ADULTS AGING SOLO WITH EARLY DEMENTIA

**DOI:** 10.1093/geroni/igad104.0015

**Published:** 2023-12-21

**Authors:** Jane Lowers, Ivree Datcher, Dio Kavalieratos, Kenneth Hepburn, Molly Perkins

**Affiliations:** Emory School of Medicine, Atlanta, Georgia, United States; Emory University, Atlanta, Georgia, United States; Emory University, Atlanta, Georgia, United States; Emory University, Atlanta, Georgia, United States; Emory University, Atlanta, Georgia, United States

## Abstract

People with Alzheimer’s and related dementias require increasing assistance with basic life activities, yet one in three lives alone (i.e., is aging solo). Drawing on a strengths-based perspective, this study seeks to identify barriers and facilitators to successful aging among adults who are aging solo with cognitive impairment or early dementia. We conducted semi-structured interviews with 15 adults aging solo with a diagnosis of cognitive impairment or early dementia (age 48-81, mean 69; 80% female; 86% white; 13% black; 7% Hispanic) and used a hybrid inductive/deductive thematic analysis approach to analyze the data. Participants identified self-advocacy, friendship networks, financial resources, and institutions (e.g., assisted living) as potential assets that could address future care needs. Participants described an identity as self-sufficient, fear of being a burden, and worries about exploitation as barriers to help-seeking. Facilitators to care-seeking from friends, neighbors, and others included a belief that helping is rewarding for others, being able to provide reciprocal favors, and framing care-seeking as self-advocacy. Conclusion: Adults aging solo with early dementia face a number of challenges but also exhibit numerous strengths and can use a diversity of skills, attitudes, relationships, and tangible resources to address increasing care needs.

